# Response of the temperate scleractinian coral *Cladocora caespitosa* to high temperature and long-term nutrient enrichment

**DOI:** 10.1038/s41598-019-50716-w

**Published:** 2019-10-02

**Authors:** Louis Hadjioannou, Carlos Jimenez, Cecile Rottier, Spyros Sfenthourakis, Christine Ferrier-Pagès

**Affiliations:** 1 Department of Biological Sciences, University of Cyprus, Nicosia, Cyprus; 2 Enalia Physis Environmental Research Centre, (ENALIA), Acropoleos 2, Aglantzia 2101, Nicosia, Cyprus; 3 Energy, Environment and Water Research Centre (EEWRC) of The Cyprus Institute, Nicosia, Cyprus; 4 Marine Department, Ecophysiology team, Centre Scientifique de Monaco, Monaco, 98000 Monaco

**Keywords:** Ecophysiology, Respiration, Animal physiology

## Abstract

Anthropogenic nutrient enrichment and increased seawater temperatures are responsible for coral reef decline. In particular, they disrupt the relationship between corals and their dinoflagellate symbionts (bleaching). However, some coral species can afford either high temperatures or nutrient enrichment and their study can bring new insights into how corals acclimate or adapt to stressors. Here, we focused on the role of the nutrient history in influencing the response of the Mediterranean scleractinian coral *Cladocora caespitosa* to thermal stress. Colonies living naturally in nutrient-poor (<0.5 µM nitrogen, <0.2 µM phosphorus, LN) and nutrient-rich (ca. 10–20 µM nitrogen, 0.4 µM phosphorus, HN) locations were sampled, maintained under the right nutrient conditions, and exposed to a temperature increase from 17 °C to 24 °C and 29 °C. While both HN and LN colonies decreased their concentrations of symbionts and/or photosynthetic pigments, HN colonies were able to maintain significant higher rates of net and gross photosynthesis at 24 °C compared to LN colonies. In addition, while there was no change in protein concentration in HN corals during the experiment, proteins continuously decreased in LN corals with increased temperature. These results are important in that they show that nutrient history can influence the response of scleractinian corals to thermal stress. Further investigations of under-studied coral groups are thus required in the future to understand the processes leading to coral resistance to environmental perturbations.

## Introduction

The health of scleractinian reef-building corals is rapidly declining, in particular due to heat wave events, which have been increasing in frequency and intensity due to global change. Elevated temperatures induce the loss of coral symbionts and/or photopigments, known as bleaching^1^. In recent years, shallow-water tropical reefs have already undergone massive bleaching events, followed by coral mortality^2–5^. Alongside deterioration in reef environment from global threats, local disturbances such as overfishing, nutrient runoff and pollution are likely to lower the resilience of corals to environmental change^6–9^. In particular, anthropogenic seawater nutrient enrichment, due to the use of chemical fertilizers or to discharges of human and animal wastes can cause shifts in trophic dynamics of coral reef ecosystems^10^, loss of coral cover and diversity^11^, increased coral diseases^12^ and susceptibility to bleaching^13^. It has also been associated to coral reef decline by disturbing the fine balance between the host and its symbiotic algae^14^. Seawater enrichment with nitrate seems to be more detrimental for corals than ammonium enrichment (reviewed by^15^), especially under imbalanced nitrogen to phosphorus ratio^13^, or in conditions, which are enriched with organic particulate matter^16^.

Despite the overall detrimental effect of nutrification on corals, it has been shown that some corals can acclimate or adapt to nutrient enriched environments^17–20^. For many Brazilian reefs, for example, there are no reports of diseases, and bleaching events have high recovery rates of corals^21,22^, despite the fact that they are both affected by high sedimentation levels^23^ and nutrient enrichment^24^. In some other cases, corals respond positively to nutrient addition, by increasing growth and metabolism^25^, especially under elevated pCO_2_^26–28^, or thermal stress^29,30^. A reduced susceptibility to bleaching was also noticed^29^, in particular in regions with small-scale upwelling^31^. Overall, these antagonistic observations suggest that more research has to be done to better understand the adaptation or acclimation of corals to nutrification. Mediterranean corals, such as the scleractinian symbiotic coral *Cladocora caespitosa*, are among the few examples of corals that can be found both in nutrient-poor (Levantine basin, Cyprus^32^) or nutrient-rich environments^20,33–35^. They are thus the perfect model to study their responses and adaptations to the nutrient levels of their living environment. In addition, they are threatened by heat waves in summer, showing several episodes of tissue necrosis (tissue degradation, peeling) and subsequent mortality^34,36–42^, due to the significant increase in sea surface temperatures (SSTs) of the Mediterranean and the Levantine Sea over the past years^43,44^. Corals, as well as other sessile organisms such as gorgonians, are key species of the Mediterranean Sea, and their mortality can have significant consequences for the ecosystem functioning and the overall biodiversity of this Sea. It is therefore urgent to understand how temperature, but also nutrient conditions, can affect their physiology and their chance to survive both global and local changes.

In this study, we have investigated the thermal tolerance of the coral *C. caespitosa* acclimated to two different nutrient environments, in order to assess the effect of nutrient supply on the response of such coral species to thermal stress. For this purpose, colonies of *C. caespitosa* were sampled in two close environments of Cyprus island, with contrasting inorganic nutrient levels: a location with low levels in inorganic nutrients, hereafter called LN (Kryo-Nero, (<0.5 µM dissolved inorganic nitrogen and 0.2 µM phosphorus), as most of the waters of the Levantine basin^32^; a location with high levels in inorganic nutrients, hereafter called HN (Liopetri > 10 µM dissolved inorganic nitrogen, 0.4 µM phosphorus), situated in front of an on-land fish hatchery and agricultural area where a large community of *C. caespitosa* thrives. We hypothesize that the main physiological traits of the coral colonies will be different between nutrient-enriched and poor conditions and that the colonies will also present a different response to thermal stress.

## Results

### Normal growth temperature (17 °C): Nutrient effect on *C. caespitosa* physiology

**Figure 1:**
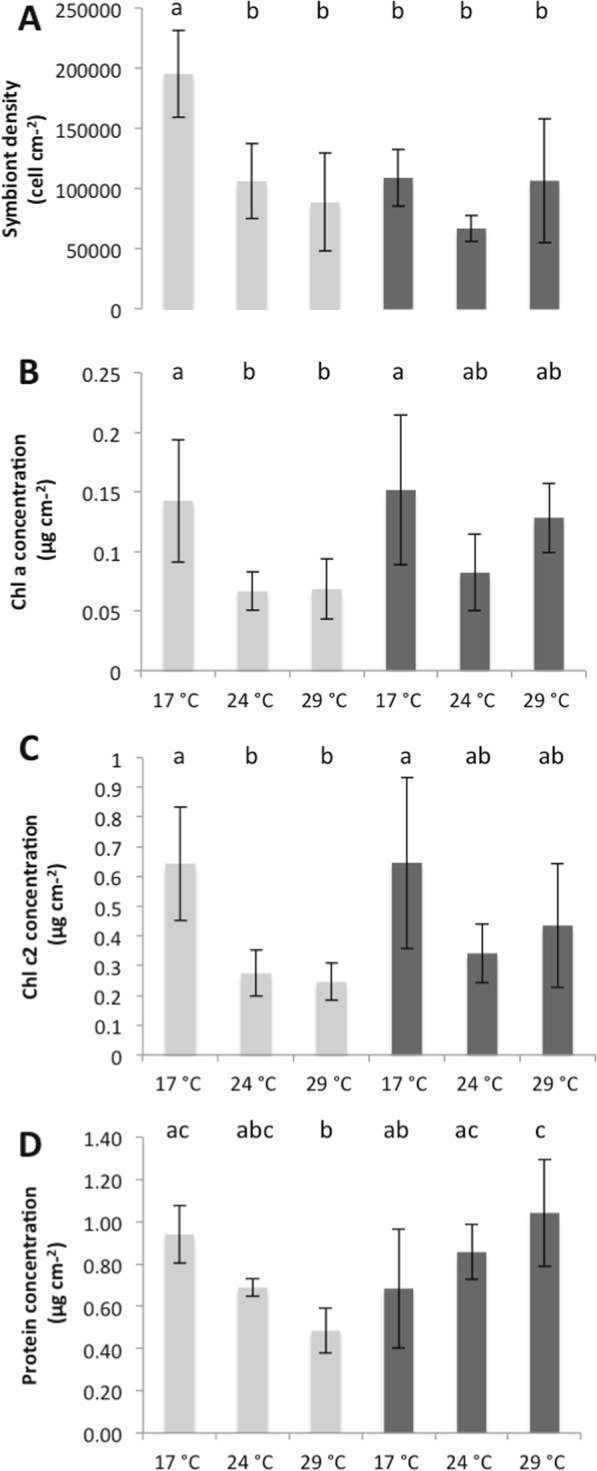
Symbiont density (**A**), concentrations in Chlorophyll-α (**B**), Chlorophyll-c2 (**C**) and protein (**D**) in nubbins maintained under low nutrient (LN, light grey) and high nutrient (HN, dark grey) conditions at different temperatures. Data represent mean ± standard deviation.

**Figure 2:**
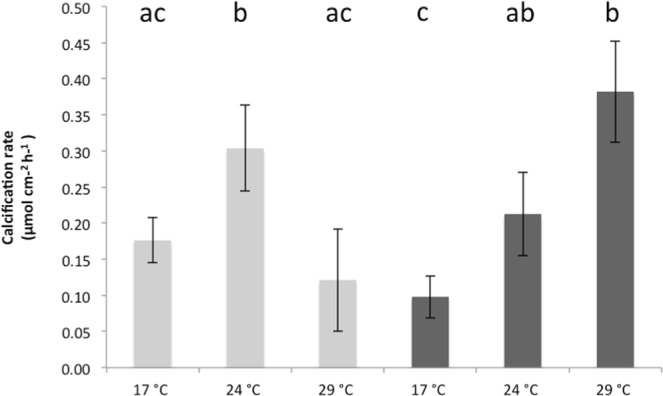
Average net photosynthesis (Pn) (**A**), respiration rates, gross photosynthesis (Pg) (**B**) and photosynthetic efficiency (Pg/symbiont) (**C**) of *C. caespitosa* under different temperatures, light intensities and nutrient levels (high (HN) and low (LN) nutrient). Data represent mean ± standard deviation.

**Figure 3:**
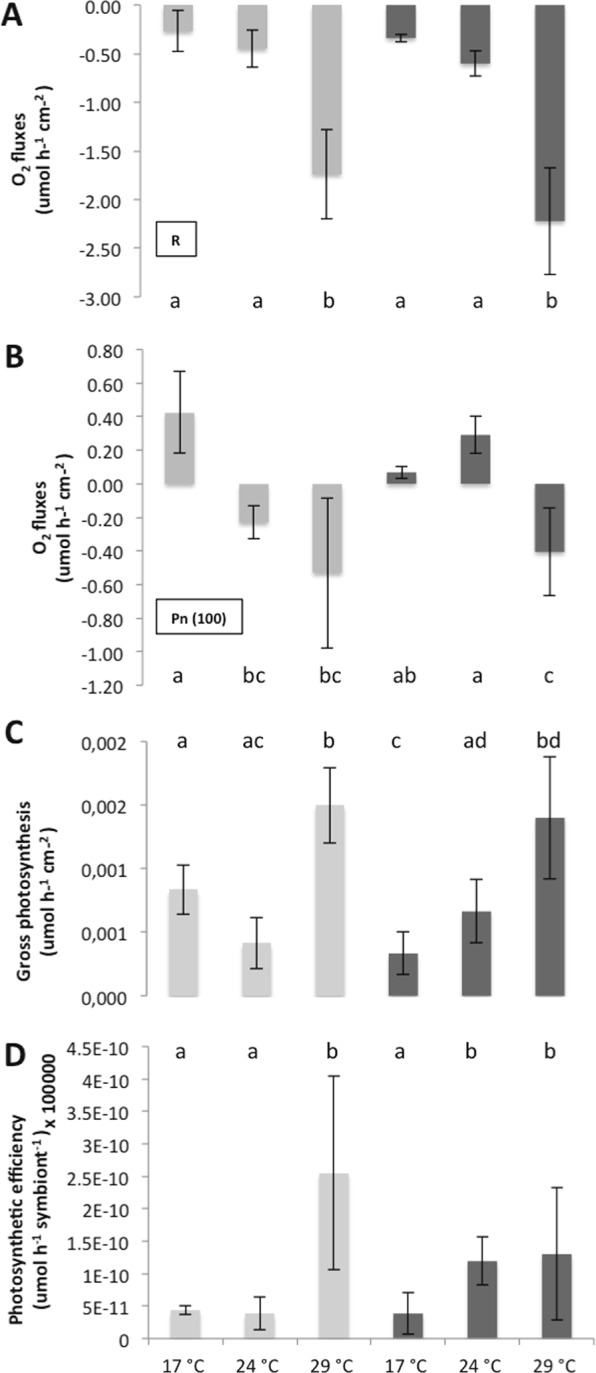
Calcification rate of *C. caespitosa* under low nutrient (light grey) and high nutrient (dark grey) levels at different seawater temperatures. Data represent mean ± standard deviation.

**Figure 4:**
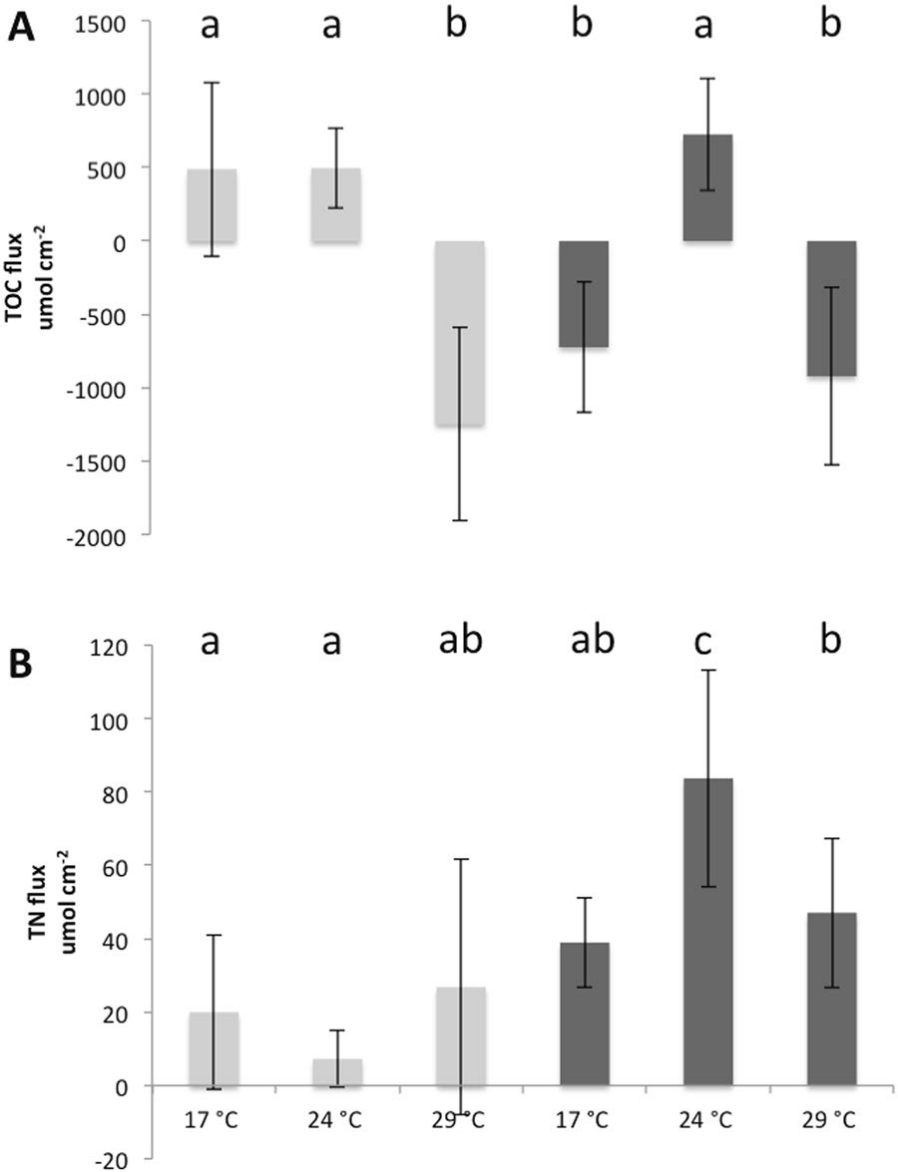
(**A**) Total organic carbon (TOC) and (**B**) total nitrogen (TN) fluxes under low nutrient (light grey) and high nutrient (dark grey) conditions, at different seawater temperatures. Data represent mean ± standard deviation.

The two-way ANOVA showed that both temperature and nutrients changed the physiology of *C. caespitosa*, with an interaction between the two parameters (Table 1). At 17 °C, nutrient enrichment significantly decreased the symbiont density per surface area (Fig. 1A; Table 1, Tukey’s test, p < 0.05), but did not significantly change the chlorophyll (a and c_2_) or protein content within the tissue of *C. caespitosa* (Fig. 1B–D; Table 1, Τukey’s test, p > 0.05). No significant changes were also observed concerning the rates of calcification (Fig. 2; Τukey’s test, p > 0.05), as well as the rates of respiration and net photosynthesis (Fig. 3A,B; Τukey’s test, p > 0.05). Gross photosynthesis was however lower in HN conditions (Fig. 3C; Τukey’s test, p < 0.01). TOC fluxes were also inversed (Fig. 4A; Τukey’s test, p < 0.01): while *C. caespitosa* released organic carbon (positive flux from the coral to the seawater) in the LN condition, it significantly took up organic carbon in HN treatment (negative flux from the coral to the seawater). This is explained by the fact that HN-corals presented lower rates of gross photosynthesis and also needed more carbon to compensate the higher levels of nitrogen input.

**Table 1:** Results of the two-way ANOVAs (p value) testing the effect of temperature and nutrient condition on the physiological parameters of *C. caespitosa*. Net photosynthesis (Pn) and gross photosynthesis (Pg) normalized to surface area (cm^−2^) or symbiont cell (symbiont), chlorophyll a (Chl a) or c2 (Chl c2) concentration, total organic carbon (TOC) and nitrogen (NTN) fluxes. NS: non significant.

	Temperature	Nutrient	Interaction
Symbiont density	<0.01	<0.05	<0.05
Chl a (μg cm^−2^)	<0.001	NS	NS
Chl c2 (μg cm^−2^)	<0.001	NS	NS
Protein (μg cm^−2^)	NS	<0.05	<0.001
Calcification	<0.001	0.053	<0.001
Pn (cm^−2^)	<0.001	NS	<0.05
Pg (cm^−2^)	<0.001	NS	<0.001
P_g_ (100) (symbiont)	<0.001	NS	<0.05
Respiration	<0.001	<0.05	NS
TOC	<0.001	NS	<0.05
TN	NS	<0.001	<0.01

### High temperatures (24 °C and 29 °C): Comparison of *C. caespitosa* physiology at low (LN) and high (HN) nutrient concentrations

There was no significant difference in the symbiont density, chl a and c_2_ content and rates of respiration between nutrient conditions at both 24 °C and 29 °C (Figs 1A–C, 3A; Τukey’s test, p > 0.05). However, at 24 °C, HN-corals presented higher rates of net and gross photosynthesis (Fig. 3B,C; Τukey’s test, p < 0.05), as well as higher rates of cell-specific photosynthesis (Fig. 3D; Τukey’s test, p < 0.05), and TN release (Fig. 4B; Τukey’s test, p < 0.001). At 29 °C, protein concentration as well as rates of calcification were also significantly higher in the HN condition compared to the LN condition (Figs 1D, 2; Τukey’s test, p < 0.001).

### Changes observed in each nutrient condition between 17 °C and high temperatures

In the LN condition, at temperatures higher than 17 °C, we observed a significant decrease in symbiont density (Fig. 1A; Τukey’s test, p < 0.05), Chl-a and chl-c_2_ concentrations (Fig. 1B,C; Τukey’s test, p < 0.05 and p < 0.01 respectively), and net photosynthesis, P_n_ (Fig. 3; Τukey’s test, p < 0.05 for 24 °C and p < 0.01 for 29 °C). Protein concentration also significantly decreased from 17 °C to 29 °C (Fig. 1D; Τukey’s test, p < 0.01). Since the dark respiration significantly increased at 29 °C when compared to 24 °C and 17 °C (Fig. 3A; Τukey’s test, p < 0.001), gross photosynthesis rates per surface area and symbiont cell were significantly higher at 29 °C compared to 17 °C (Fig. 3C,D; Τukey’s test, p < 0.001 and < 0.01 respectively). Finally, calcification rates, measured through total alkalinity, significantly increased from 17 °C to 24 °C (Fig. 3; Τukey’s test, p < 0.05), before decreasing again to the initial value at 29 °C (Fig. 3; Tukey’s test, p < 0.001). While there were no significant changes in the TN fluxes with temperature conditions, TOC fluxes were inversed between 17 °C-24 °C and 29 °C (Fig. 4; Τukey’s test, p < 0.001): *C. caespitosa* released TOC at 17 °C and 24 °C while it took up TOC at 29 °C.

In the HN condition, there was no significant change in symbiont density (Fig. 1A; Τukey’s test, p > 0.05), and chl a and c2 levels (Fig. 1B,C; Τukey’s test, p > 0.05;) with increased seawater temperature. Some parameters significantly increased between 17 °C and 29 °C: protein concentration (Fig. 1D; Τukey’s test, p < 0.05), respiration rates (Fig. 2A; Τukey’s test, p < 0.001), P_g_ (Fig. 2C; Tukey’s test, p < 0.001), rates of photosynthesis per symbiont cell (Fig. 2D; Tukey’s test, p < 0.05) and rates of calcification (Fig. 3; Τukey’s test, p < 0.001). The two last parameters started to significantly increase at 24 °C (Figs 3, 4D; Τukey’s test, p < 0.05 and p < 0.05 respectively). On the contrary, P_n_ was significantly lower at 29 °C compared to the other temperatures (Fig. 2B; Tukey’s test, p < 0.05). Finally, TOC and TN fluxes were significantly higher at 24 °C when compared to 17 °C (Fig. 4A,B; Τukey’s test, p < 0.001) and 29 °C (Fig. 4A,B; Τukey’s test, p < 0.001).

## Discussion

Although many coral species are vulnerable to increased sea surface temperature and/or nutrification^45–47^, some may acclimate or even adapt to these stressors, at both the physiological and molecular levels (i.e.^48^). For example, thermal history led to acclimation in several coral species^49–53^ (see^5^ for an alternative view) and some corals are able to grow in nutrified or eutrophic environments^17,54^. Although the above studies have highlighted the importance of understanding the flexibility of coral responses to environmental stressors, most of them have focused on the acclimation to high temperature conditions rather than nutrification. It is however important to understand the ability of different coral species to acclimate to high nutrient conditions, as this is going to affect many reefs in the future, due to the increasing urbanization of many coastal areas (e.g.^8^). The present study is thus one of the few that has focused on experimentally testing the effect of the long-term nutrient history on the bleaching susceptibility of a scleractinian coral species^47,55^. Mediterranean corals such as *C. caespitosa* are good examples of coral species able to thrive both in oligotrophic and nutrient-enriched, environments. In addition, they experience large temperature variations between summer and winter conditions^56^. The results of this study are important because they show that the nutrient history can influence the response of some scleractinian corals to thermal stress and therefore have implications for the understanding of the bleaching process and coral resilience^57,58^. We observed that colonies acclimated to very high levels of dissolved inorganic nutrients didn’t bleach more and even maintained higher rates of net and gross photosynthesis and higher protein content than non-enriched corals at elevated temperatures. Our results provide novel insights into the particular resilience of Mediterranen corals to nutrification, as also observed in some particular occasions with tropical corals^17,54^. They suggest that further investigation of under-studied coral groups are needed in the future to understand the processes leading to such coral resilience to environmental perturbations.

Colonies of *C. caespitosa* maintained under low inorganic nutrient concentrations, but fed twice a week with *Artemia salina* prey at repletion, presented a response to thermal stress similar to many tropical and temperate coral species^37,46,59,60^. Temperature increase induced a significant decrease in both symbiont density and areal chlorophyll content of *C. caespitosa*, followed by a decrease in the net photosynthesis measured at the *in situ* irradiance of 100 µmole photons m^−2^ s^−1^ (bleaching). As a consequence of the decreased autotrophic energy input and increased respiratory needs at high temperatures, the protein content of the coral tissue, which is a proxy of biomass, continuously decreased during the elevation in temperature. Such decrease occurred despite the fact that corals stopped releasing organic carbon and even started to take up the small amount of carbon available in seawater at 29 °C (Fig. 4). Bleaching also occurred while coral colonies were fed twice a week with *Artemia salina* nauplii. Feeding has been shown to decrease the bleaching susceptibility of tropical coral species^61–63^ but did not avoid bleaching in *C. caespitosa*, likely because it is an heterotrophic species with high energetic requirements. Mortality or bleaching of *C. caespitosa* has thereby been recorded in different locations of the Mediterranean Sea^38,39,64,65^ but also in Cyprus in 2012, concurring with temperature anomalies^42^. A similar effect of high temperature on *C. caespitosa* was observed in laboratory thermal stress experiments^37^. Despite significant bleaching, calcification rates were boosted under high temperature conditions. This is in agreement with *in situ* observations in the North-West Mediterranean Sea showing higher growth rates of *C. caespitosa* in summer, compared to almost no growth in winter, at temperatures of 12 °C^66^. The growth of *C. caespitosa* in a previous thermal stress experiment^37^ was also significantly enhanced during the first 3 weeks of temperature increase, contrary to another Mediterranean coral, *Oculina patagonica*, whose growth was rapidly impacted by thermal stress^37^. In tropical corals, the thermal optimum for calcification generally occurs between 26 °C and 28 °C, after which there is an inverse temperature dependency^67,68^. Calcification of *C. caespitosa* may follow the same trend, at least until the energetic reserves in coral tissue are able to sustain such high growth rate.

One of the major observations of this study is the particular resistance of *C. caespitosa* to long-term nutrification. The same can be observed in other parts of the Mediterranean Sea, such as close to the city of La Spezia (North West Mediterranean Sea), where many colonies also thrive next to a river mouth in a nutrient rich environment^66^. At the *in situ* temperature of 17 °C, high nitrogen supply did not increase the symbiont density, which is in contrast with many tropical corals^68,69^. In these later corals, increased symbiont density may even lead to a decrease in rates of photosynthesis and calcification^70–72^. It has to be noticed that *C. caespitosa*, under natural conditions, can afford relatively high symbiont densities, particularly in the North-West Mediterranean Sea, where it can host more than 2 and up to 6 × 10^−6^ zooxanthellae cm^−2^ ^37,73^. The lack of nitrogen enhancement of symbiont growth can be partly due to the fact that symbionts are already nutrient-repleted, due to the particular heterotrophic nature of *C. caespitosa*, which mainly feed on planktonic prey throughout the year^74^. We also observed at 17 °C, under high-nutrient condition, an adjustment with lower symbiont density, but higher cell-specific photosynthetic pigments compared to corals maintained under low-nutrient condition. All together, the areal photosynthetic pigment concentration, as well as the rates of net photosynthesis did not change between nutrient-enriched and poor conditions, which did not affect the rates of calcification. Nutrification also promoted the uptake of (dissolved and particulate) organic carbon contained in seawater by *C. caespitosa*, suggesting that the corals had to counterbalance the high nitrogen input by acquiring more carbon from seawater.

In the tropics, chronic enrichment in dissolved inorganic nutrients, especially nitrogen, has direct but also indirect effects on corals (reviewed in^14^). It enhances the prevalence and severity of coral disease^12,55^, leads to imbalanced N:P ratios within the coral tissue^13,75^, and increases coral bleaching susceptibility, especially under a combined enrichment in nitrate and particulate organic matter^16^. At the ecosystem level, it mainly increases the density and productivity of macroalgae, which can overgrow and replace corals^76^, alter the coral microbial communities and interfere with recruitment of planulae by allelopathic interactions^77,78^. The success of *C. caespitosa* in shallow eutrophic areas of the Mediterranean Sea can thus partly rely on the lack of competition with algae, due to water turbidity or algal grazing by sea urchin^35^. Although *C. caespitosa* banks can be observed in Spain in the middle of a high algal coverage of *Dictyopteris polypodioides*, *Halimeda tuna*, *Cystoseira sauvageauana* and *Cystoseira compressa*^35^, the algae were indeed never observed overgrowing coral colonies. Finally, the heterotrophic nature of *C. caespitosa* can explain its presence in eutrophic environments^74^. The same observation was made in tropical areas, where the increased productivity of nutrient enriched waters has benefited corals with a high heterotrophic capacity^11,79,80^.

Another major observation of this study is that nutrification did not induce enhanced bleaching of *C. caespitosa* under high temperatures compared to control corals and even maintained higher rates of photosynthesis at 24 °C, as well as a higher protein content at 29 °C. Although moderate inorganic nitrogen supply (ca. 1–3 µM) has been shown to promote coral growth and metabolism^17,81^, in particular under elevated pCO_2_^25–27^ or thermal stress^30^, other studies on tropical corals have also suggested that elevated inorganic nitrogen levels may impact corals by decreasing their thermal thresholds for bleaching. Nitrogen addition indeed tends to enhance symbiont growth inside the coral host tissue and increase oxidative stress^13,55,82–84^. To reconcile these two opposite observations, Wiedenmann *et al*.^13^, as well as some other studies^75,85^ demonstrated that an imbalance N:P ratio was the key factor explaining coral bleaching. A condition where phosphorus is in limited amount while nitrate is fully available indeed promotes coral bleaching^13,75,85^. In this study, while the seawater N:P ratio was high and should have induced bleaching in *C. caespitosa*, the contrary was observed. A plausible explanation is that the internal N:P ratio of the coral tissue was not imbalanced, due to the provision of heterotrophic food to the coral colonies, which may have delivered large amounts of organic phosphorus to the coral^74^. Heterotrophy may have also avoided carbon limitation of the symbionts under high nutrient supply^13,63^. Such carbon limitation has often been reported in coral-dinoflagellate symbiosis^86,87^, enhancing bleaching under thermal stress^63,87,88^. Since the physiological traits of the coral host are partly shaped by the dominant symbiont type present within its tissues^89^, we also suggest that the symbionts of *C. caespitosa* have particular adaptation to nutrient enrichment and can provide ecological advantages to *C. caespitosa* in nutrient-rich conditions. Symbionts in *C. caespitosa* belong to formerly clade B (now *Breviolum* sp.), which is common in the Mediterranean temperate and subtropical regions^90–92^. In the light of these observations, more studies are needed to fully understand the interactions between organic and inorganic nutrients on the resistance of corals to thermal stress, in particular by taking into account how external nutrients modify the internal C:N:P ratio of coral tissue. In addition, the response of corals to environmental changes may be light dependent, as shown in recent studies^93,94^. This experiment was performed in late autumn/winter on samples that were acclimated to relatively low light levels (100 µmol photons m^−2^ s^−1^). The experiment should therefore be repeated during the summer season, when irradiance can be 3 times higher.

*Cladocora caespitosa* is an emblematic coral of the Mediterranean Sea, and its conservation is an important concern now that its bioconstructions are endangered by the climate change effects^35^. A better knowledge of its response to environmental stressors is thus needed to further understand how this species can be preserved. This study conclusively demonstrates that the long time scale acclimation to high nutrient levels can reduce the bleaching susceptibility of *C. caespitosa* and has not necessarily adverse effects on its growth. This is maybe due to the high heterotrophic capacities of the coral host, which can maintain a balanced C:N:P ratio within the tissues and counterbalance the nutrient-enhancement of symbiont growth. However, this coral model need more in depth studies to fully understand the different acclimation or adaptation ways to eutrophication.

## Materials and Methods

### Study sites and sample collection

**Figure 5:**
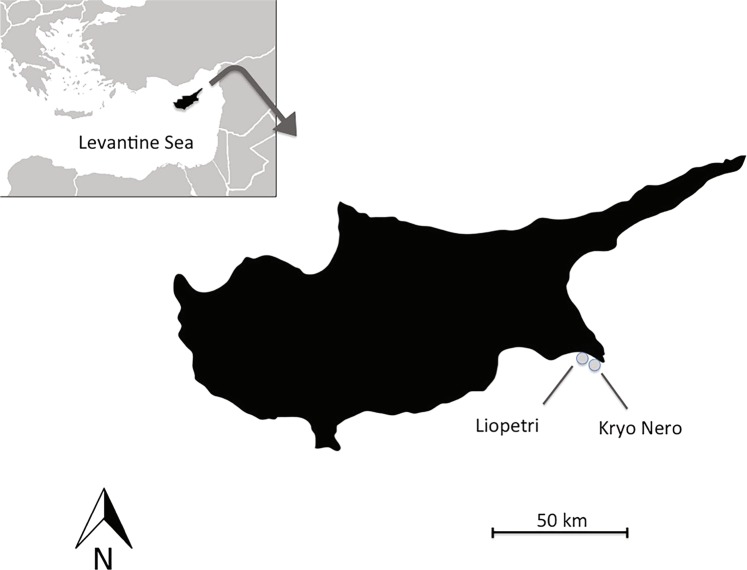
Location of field-sites in SE Cyprus.

Coral colonies originated from two close areas in Cyprus, both holding > 100 colonies of *C. caespitosa* at very shallow depths (<4 m). ‘Kryo Nero’ site (i.e. nutrient-poor site, LN), is found on the coast of Ayia Napa village in the South-east of Cyprus (34°58.949′N, 34°1.014′E). ‘Liopetri’ site (nutrient-enriched site, HN) lies approximately 10 km west of ‘Kryo Nero’ right in front of a small on-land fish hatchery and very close to a large agricultural area (34°57.537′N, 33°53.755′E) (Fig. 5).

Prior to the experiments, water samples were collected from both locations (35 times between 2012–2015 from Liopetri; 12 times between 2014–2015 from Kryo Nero) and analyzed to determine inorganic nutrient concentrations using standard spectrophotometric methods^95^. Inorganic nutrient analyses showed significantly higher concentrations at Liopetri than in Kryo Nero. Mean nutrient concentrations at Liopetri were 1232 μg L^−1^ or 19.87 µM for nitrate (NO_3_^−^), 92 μg L^−1^ or 5 µM for ammonium (NH_4_^+^) and 24 μg L^−1^ or 0.24 µM for phosphate (PO_4_^3−^). At Kryo Nero, mean concentrations equaled 74 μg L^−1^ or 1.2 µM NO_3_^−^, 13 μg L^−1^ or 0.72 µM NH_4_^+^, 12 μg L^−1^ or 0.12 µM PO_4_^3−^ (Supplementary Fig. S1). The particulate organic carbon (POC) and nitrogen (PON) content of the water was also analyzed using an elemental analyzer (Shimadzu). POC concentrations were equal to 27.6 ± 5.7 µM and 25.5 ± 2.5 in Kryo Nero and Liopetri respectively. PON concentrations ranged from 1.4 ± 0.4 µM in Kryo Nero to 1.8 ± 0.14 µM in Liopetri. Both levels were not significantly different between locations and in agreement with previous measurements for the Mediterranean Sea^96^.

Coral fragments (of 6–8 polyps) were collected from 36 large colonies at Liopetri and Kryo Nero, end of November 2015. They were identified, kept in separated bags containing the original seawater and rapidly transported to the aquarium system of the Centre Scientifique de Monaco (CITES no CY/exp/005/2015). Here, each fragment was divided in two smaller fragments of 3–4 polyps, making a total of 72 fragments, which were distributed into 12 tanks, so that each tank contained 6 different original colonies. All tanks were maintained at the seawater temperature at the time of collection (17 °C). Six tanks were maintained under low nutrient condition (ca. 0.5 µM NO_3_^−^, 0.1 µM NH_4_^+^ and 0.2 µM PO_4_^3−^) whereas the other six received high nitrogen levels (6–7 μM NO_3_^−^ and 5–6 μM NH_4_^+^). These concentrations were lower than the mean *in situ* concentrations, but where applied continuously to the corals for the 6 weeks experiment. Nutrient enrichment was thus performed using a peristaltic pump, which continuously supplied the experimental tanks with a solution of NO_3_^−^ and NH_4_^+^ at a rate of 15 ml h^−1^, together with a 12 L h^−1^ seawater flow-through. Nutrient concentrations were monitored twice a week with an auto-analyzer (Alliance Instrument, France), according to Tréguer and Le Corre (1975)^97^. Light (100 ± 10 μmol photons m^−2^ s^−1^, with a 12:12 h photoperiod) was provided by HQI lamps and set up to the mean daily irradiance received by the corals at the time of collection (daily photon flux density of 4 mol m^−2^). It was measured using a spherical quantum sensor (LiCor LI-193, Lincoln, NE, USA). As *C. caespitosa* is a mixotrophic/heterotrophic species, colonies were fed twice a week with nauplii of *Artemia salina*. This ensured to have the same level of heterotrophic feeding but a different autotrophic level linked to the two inorganic nutrient conditions.

### Experimental setup

Corals were kept three weeks under the two nutrient conditions and at 17 °C (control). Two aquaria per nutrient condition were kept as control while seawater temperature was slowly increased (0.5 °C per day) in two other aquaria to 24 °C and the last two aquaria to 29 °C. Once the last two aquaria reached 29 °C, corals were all maintained for 10 days before the physiological measurements described below were performed. While 17 °C corresponds to the temperature at the time of collection, 24 °C and 29 °C represent respectively the mean annual temperature in Cyprus and the mean maximal temperature recorded in summer times using a Star-Oddi starmon mini temperature logger.

### Measurements

#### Calcification and release of organic carbon and nitrogen

Calcification rates were assessed using the alkalinity anomalous technique/principle, according to Smith and Kinsey (1978)^98^. Six nubbins from each condition (3 per tank) were placed in separate sealed containers with 350 mL of 0.22 μm-filtered seawater (FSW). An extra container with only FSW was also incubated to serve as control. All containers were placed in a water bath at the right temperature (17 °C, 24 °C, 29 °C) and light and incubated for 6 hours. Stirring was applied by magnetic stir bars. At the beginning and end of the incubation period, three seawater samples (50 mL) were collected from each container and transferred in borosilicated vials. The TA was immediately measured in duplicate by automatic titration using a Metrohm Titrando 888 following Dickson *et al*.^99^.

The same coral nubbins were used to estimate the total organic carbon (TOC) and nitrogen fluxes (TN) with the use of Shimadzu TOC-L analyser, according to the established beaker incubation technique (e.g.^100^). Briefly, corals were transferred without aerial exposure into acid-washed and seawater-rinsed 250 ml glass beakers filled with 0.2 µm filtered seawater. Three control beakers containing only seawater were also prepared. All beakers were placed in a water bath and incubated for 6 h as described above. After 6 h, corals were removed from the incubation beakers and kept for surface determination. Before and after incubations, seawater subsamples were drawn by sterile syringe from the thoroughly homogenised incubation media to quantify TOC and TON concentrations. Subsamples were transferred into pre-combusted (450 °C, 5 h) glass vials, acidified with phosphoric acid (20%, 250 μl) to pH < 2 and kept frozen (−20 °C) until analysis.

#### Photosynthesis/respiration

Rates of net photosynthesis (P_n_) and respiration (R) were measured using six nubbins per condition (three per tank). Each nubbin was placed in a temperature-controlled airtight chamber filled with ~50 ml of 0.45 μm-FSW, equipped with optodes (OCY-4 micro, PreSens, Germany), and continuously stirred using magnetic stirrers. The optodes were calibrated before each treatment using nitrogen gas (N_2_) and air saturated water for 0% and 100% oxygen saturation values respectively. Measurements were performed during 15 minutes initially at 100 μmol photons m^−2^ s^−1^, and then 20 minutes in total darkness. Rates of gross photosynthesis (P_g_) were calculated by adding R to P_n_. Rates of cell photosynthesis (P_g_/zoox) were calculated by normalizing P_g_ to symbiont density. Each rate was expressed per polyp surface area (µmol O_2_ h^−1^ cm^−2^) or per symbiont cell (µmol O_2_ h^−1^ symbiont cell^−1^) according to Rodolfo-Metalpa *et al*.^37^. Samples were frozen for later determination of tissue parameters (symbiont, chlorophyll concentration, and protein concentration).

Tissue parameters were determined according to Hoogenboom *et al*.^73^. Coral tissue was removed from the skeleton with an airbrush, using 0.45 μm filtered seawater and homogenized with a potter tissue grinder. A 1 mL sub-sample was used to determine symbiont density with a Beckman coulter counter (France). Protein content was assessed in another 1 mL sample according to Smith *et al*.^101^ by the use of a BCAssay Protein Quantification Kit (Uptima, Interchim) and a Xenius® spectrofluorometer (SAFAS, Monaco). In order to measure Chlorophyll-a concentration, the remaining 5 mL sub-sample was centrifuged at 8000 g for 10 min at 4 °C. After removing the supernatant, symbionts were resuspended into 5 mL acetone and placed at 4 °C overnight. Chlorophyll a and c_2_ concentrations were determined following the method of Jeffrey and Humphrey (1975)^102^ by the use of a spectrophotometer (Safas, Monaco). Data were normalized to the surface area (cm^2^). The main Symbiodiniacae genotype hosted by *C. caespitosa* in each location was checked according to the protocol of Santos *et al*.^103^. Symbionts from both sampling sites belong to clade B.

### Statistical analyses

Two-way analysis of variance (ANOVA) was used to compare TA, TOC, TN, P_n_, P_g_, P_g_/zoox, symbiont density, chlorophyll-a/chlorophyll-c2 and protein concentrations between nutrient conditions and temperatures. When significant interaction effects were detected, Tukey’s HSD multiple comparison tests were conducted to examine the differences. All data were checked prior to analyses for normal distribution and were log-transformed when required. All analyses were computed using PAST statistical package^104^. Comparisons with p < 0.05 were considered significant.

## Supplementary information

Supplementary information

## Data Availability

All material, data and associated protocols are available from the authors.
