# Supplementary material for: Response of the temperate scleractinian coral Cladocora caespitosa to high temperature and long-term nutrient enrichment
Source: Sci Rep. 2019 Oct 2;9:14229. doi: 10.1038/s41598-019-50716-w (PMC6775152; doi:10.1038/s41598-019-50716-w)
Supplement: Supplementary file 1 — Supplementary information [file 41598_2019_50716_MOESM1_ESM.docx]

**Response of the temperate scleractinian coral *Cladocora caespitosa* to high temperature and long-term nutrient enrichment.**

Louis Hadjioannou^1, 2*^, Carlos Jimenez ^2, 3^, Cecile Rottier ^4^, Spyros Sfenthourakis^1^, Christine Ferrier-Pagès ^4^

^1^ Department of Biological Sciences, University of Cyprus, Nicosia, Cyprus

^2^ Enalia Physis Environmental Research Centre, (ENALIA), Acropoleos 2, Aglantzia 2101, Nicosia; Cyprus

^3^ Energy, Environment and Water Research Centre (EEWRC) of The Cyprus Institute, Cyprus

^4^ Marine Department, Ecophysiology team, Centre Scientifique de Monaco, Monaco, 98000 Monaco

**Supplementary material**

Figure S1. Nutrient concentrations from coral sites.
